# Cinchonia in Menorrhagia—Case

**Published:** 1859-08

**Authors:** Robert Battey

**Affiliations:** Rome, Georgia


### ARTICLE VI.

*Cinchonia in Menorrhagia—Case.* By ROBERT BATTEY, M.D., Rome, Georgia.

April 25th, 1857, Amanda, negro woman, aged 25, field hand, mother of one child 8 years old, complains of menorrhagia of seven years standing—at times so profuse as to excite alarm for her safety. Menses irregular; sometimes misses one term; of late comes on every 21st day, with some degree of regularity; discharge clotted, free and debilitating; continues 3 to 4 days; usual time in health, 2 to 3 days; complains of hypogastric and sacral pains; also shooting down the innersides of the thighs; no considerable leucorrhœa; ordinarily no sense of weight in the pelvic region. The touch shows no malposition of the uterus, nor does the speculum reveal any abnormal condition, save a slight palor of the mucous membrane. Prof. Simpson's sound did not penetrate the canal of the cervix more than half an inch; os uteri small but healthy; the length of the organ, as determined by the simultaneous touch from above and below, was not materially augmented. The long standing and severity of the symptoms would lead us to expect more tangible evidences of diseased uterus.

Patient was directed to wear short drawers of red flannel, use the cold hip bath daily, with friction after, by means of a rough crash towel; bowels to be kept regular by using the following "ferro saline" solution of Dr. Dunglison:

℞.—Sulphate of magnesia, one ounce,
Bitartrate of potassa, two drachms,

Sulphate of Iron, ten grains,

Water, two pints.

Make a solution and direct a wine-glassful to be taken every morning on getting out of bed. As a tonic the following mixture:

℞.—Sulphate of cinchonia, two drachms,
Elixir of vitriol, two drachms,
Syrup of ginger, two ounces,
Water, six ounces.

Make a solution and direct a teaspoonful thrice daily.

May 10th.—Reports herself greatly improved—catamenial period during the week was passed over with more than ordinary ease and comfort—has not absented herself from the field a single day—bowels in good condition. Treatment extended four weeks longer.

Amanda continued improving, and was discharged upon the 1st of July as well. At her menstrual period in August, while engaged in pulling fodder; she had a return of the pain, with some hemorrhage, not sufficient, however, to require the service of her physician. She resumed the use of the cinchonia for some four or six weeks, and has had no return of her menorrhagia since, now near two years.

The query is perhaps, worthy of record, as suggested by this case—has not the *antiperiodic* property of cinchonia, (or quinia,) a more potent influence in the cure of what may perhaps, not inaptly be termed a *menorrhagia from habit*, than the mere *tonic* influence of the drug? It is quite sure, that these remedies (cinchonia and quinia,) are eminently serviceable in treating the chronic uterine ailments, involving disordered menstruation. The patient in question, during the fall previous, had used iodide of iron with advantage to her general health, but with no manifest influence upon her menorrhagia.

